# Fecal chromogranins and secretogranins are linked to the fecal and mucosal intestinal bacterial composition of IBS patients and healthy subjects

**DOI:** 10.1038/s41598-018-35241-6

**Published:** 2018-11-14

**Authors:** Johanna Sundin, Mats Stridsberg, Julien Tap, Muriel Derrien, Boris Le Nevé, Joël Doré, Hans Törnblom, Magnus Simrén, Lena Öhman

**Affiliations:** 1 Institute of Medicine, University of Gothenburg, Gothenburg, Sweden; 2 Institute of Biomedicine, University of Gothenburg, Gothenburg, Sweden; 3 Department of Medical Sciences, Uppsala University, Uppsala, Sweden; 4 Danone Nutricia Research, Department of Innovation, Science and Nutrition, Palaiseau, France; 5 French National Institute for Agricultural Research (INRA) MetaGenoPolis, Jouy-en-Josas, France; 6 Centre for Functional Gastrointestinal and Motility Disorders, University of North Carolina at Chapel Hill, Chapel Hill, NC USA

## Abstract

Altered fecal levels of chromogranins (Cg) and secretogranins (Sg) are demonstrated in irritable bowel syndrome (IBS), but their role in IBS pathophysiology remains unknown. This study aimed to determine if granins are associated with bacterial composition, immune activation and IBS symptoms. Protein levels of fecal granins (CgA, CgB, SgII and SgIII) were analysed with immunoassays. Mucosal mRNA expression of granins, TPH1 and immune markers were evaluated with RT-qPCR. 16S rRNA gene sequencing was performed on fecal and mucosal bacteria. The intestinal granin profile, based on fecal protein levels and mucosal mRNA expression, could not discriminate between IBS patients (n = 88) and healthy subjects (HS, n = 33). IBS patients dominated by high fecal or mucosal granin levels, respectively, did not differ in symptom or immune profiles. Fecal-dominated and mucosal-dominated granin clusters of IBS patients and HS, demonstrated separate fecal and mucosal bacterial profiles and high fecal abundance of granins were associated with a less diverse bacterial composition and the *Bacteroides* enterotype. The intestinal granin profiles of IBS patients and HS are linked to the intestinal bacterial composition, diversity and enterotypes. These findings suggest that granins may be one of several host-produced factors regulating the microbiota composition of the intestine.

## Introduction

Irritable bowel syndrome (IBS) is a chronic disorder in which patients suffer from gastrointestinal symptoms such as pain/discomfort, bloating and altered bowel habits^1^. The pathophysiology of IBS is multifactorial including abnormalities in the gut microbiota, gut-brain interactions, the enteric nervous system (ENS) as well as the immune system^2–6^. Previous studies have shown altered intestinal protein levels of chromogranins and secretogranins (granins) in IBS patients compared to healthy subjects^7–10^. However, it is currently not known whether granins are part of IBS aetiology, or linked to IBS symptoms.

Chromogranins and secretogranins are signalling proteins that act as hormones and through their release both into the bloodstream and lumen facilitate communication between intestinal immune cells and the terminal axons of enteric neurons and enteroendocrine cells (EEC). Chromogranin and secretogranin producing cells are located in the mucosa along the gastrointestinal tract and in addition to EECs, which are the main producers, chromogranins and secretogranins are also secreted by immune and nerve cells^11–13^.

Chromogranins and secretogranins share physiochemical properties including their acidity, ability to bind calcium, tendency to form aggregates and their multiple cleavage sites. Therefore, together they form the granin family, which include chromogranin A (CgA), chromogranin B (CgB), secretogranin II (SgII) and secretogranin III^14^.

While little is known about secretogranins, the synthesis of chromogranins is regulated by serotonin, which in turn is regulated by gut microbiota, at least in mice^15,16^. Therefore, even though the interplay between gut produced signalling proteins such as chromogranins and secretogranins and bacterial homoeostasis is not fully elucidated, the link to serotonin suggests that these proteins are important factors in the regulation of gut health. A healthy gut microbiota composition is thought to have a high bacterial diversity^17^. While some studies have shown that the bacterial diversity in the intestine is reduced in subsets of IBS patients^5,18,19^, it is not yet elucidated whether lower bacterial diversity is causal or consequent to IBS physiopathology.

In addition to neuro-immune interactions, granins have the ability to act pro-inflammatory and anti-inflammatory, at least *in vitro*, and may therefore have an indirect effect on bacterial growth^20^. Furthermore, in the lumen granins are in direct contact with the bacteria and *in vitro*, granin derived substances directly inhibit the growth of microorganisms including bacteria such as for example *Micrococcus luteus* and *Bacillus megaterium* as well as fungi including the yeasts *Saccharomyces cerevisiae* and *Candida albicans*^21,22^. Thus, the ability of granins to *in vitro* affect bacterial growth both directly and indirectly suggests a potential link between intestinal bacterial composition and granins.

Hence, we hypothesized that chromogranins and secretogranins are altered in IBS patients, or subgroups thereof, and may have the potential to affect gastrointestinal and psychological symptoms, activate the intestinal immune system and regulate the growth of the gut microbiota. In this study, we therefore aim to explore the link between fecal protein levels and mucosal mRNA expression of chromogranins and secretogranins, IBS symptom profiles, immune activation and fecal and mucosal intestinal bacterial composition in a well-characterized IBS cohort and compared it to healthy subjects.

## Results

### Clinical characteristics of IBS patients

In total, 88 well-characterized IBS patients (disease duration 10 (5–20) years) and 33 healthy subjects were included in the study. Among IBS patients, 27 were defined as IBS-C, 31 as IBS-D and 30 as IBS-nonCnonD (Table 1). Fecal samples for protein analysis and bacterial DNA analysis, as well as biopsies for mucosal mRNA expression analysis of granins were collected from all study subjects. Additionally, from this study cohort, 30 IBS patients and 14 healthy subjects also provided biopsies for bacterial DNA analysis. Thirteen patients were defined as having PI-IBS after a self-reported bout of gastroenteritis (Table 1). IBS patients were older than healthy subjects (*p* < 0.01), while there were no gender differences between the groups (Table 1). HAD scores were significantly higher in IBS patients compared to healthy subjects (*p* = 0.0001, Table 1). Fecal calprotectin protein levels did not differ between IBS patients and healthy subjects (11 (10–45) *vs*. 18 (10–47) mg/kg).

**Table 1:** Clinical characteristics median (range 25–75%) of irritable bowel syndrome (IBS) patients and healthy subjects.

	IBS	HC	*p*-value*
	(n = 88)	(n = 33)	
Sex (F/M)	54/34	21/12-dec	*0*.*84*
Age (years)	35 (28–44)	27 (25–35)	***0***.***01***
IBS-SSS	249 (200–363)	N/A	
HADS score	12 (8–19)	7 (4–10)	***0***.***0001***
VSI score	42 (31–55)	N/A	
Subtype (IBS-D/IBS-C/nonCnonD)	27/31/30	N/A	
PI-IBS	13	N/A	

Abbreviations: IBS-C = Constipation predominant IBS, PI-IBS = Post infectious IBS, IBS-D = Diarrhoea predominant IBS, IBS-SSS = IBS Symptom Severity Scale, HADS = Hospital Anxiety and Depression scale, VSI = Visceral sensitivity index scale, *Mann Whitney U Test between groups.

### IBS patients and healthy subjects have similar intestinal granin profiles

Univariate analysis showed no difference in fecal levels or mucosal expression of CgA, CgB, SgII or SgIII between IBS patients and healthy subjects (Supplementary Table 1). There were no differences between PI-IBS and non-PI-IBS patients in fecal protein levels of granins (CgA, CgB, SgII or SgIII) or mucosal expression of CgB, SgII or SgIII. However, mucosal CgA expression was slightly higher in PI-IBS patients (0.15 (0.11–0.25)) as compared to non-PI-IBS patients (0.10 (0.06–0.16), *p* < 0.05). Further, there were no correlations detected between age and any of the fecal or mucosal granins (Fecal protein levels: CgA r = 0.07, CgB r = 0.04, SgII r = 0.04 and SgIII r = 0.07; Mucosal expression: CgA r = 0.07, CgB r = 0.06, SgII r = −0.04 and SgIII r = 0.05). In both IBS patients and healthy subjects the protein levels of fecal CgA, CgB, SgII and SgIII and mucosal mRNA expression of CgA, CgB and SgIII were highly correlated with each other, while the mucosal mRNA expression of SgII did not correlate with the other mucosal granins (Supplementary Table 2).

**Figure 1:**
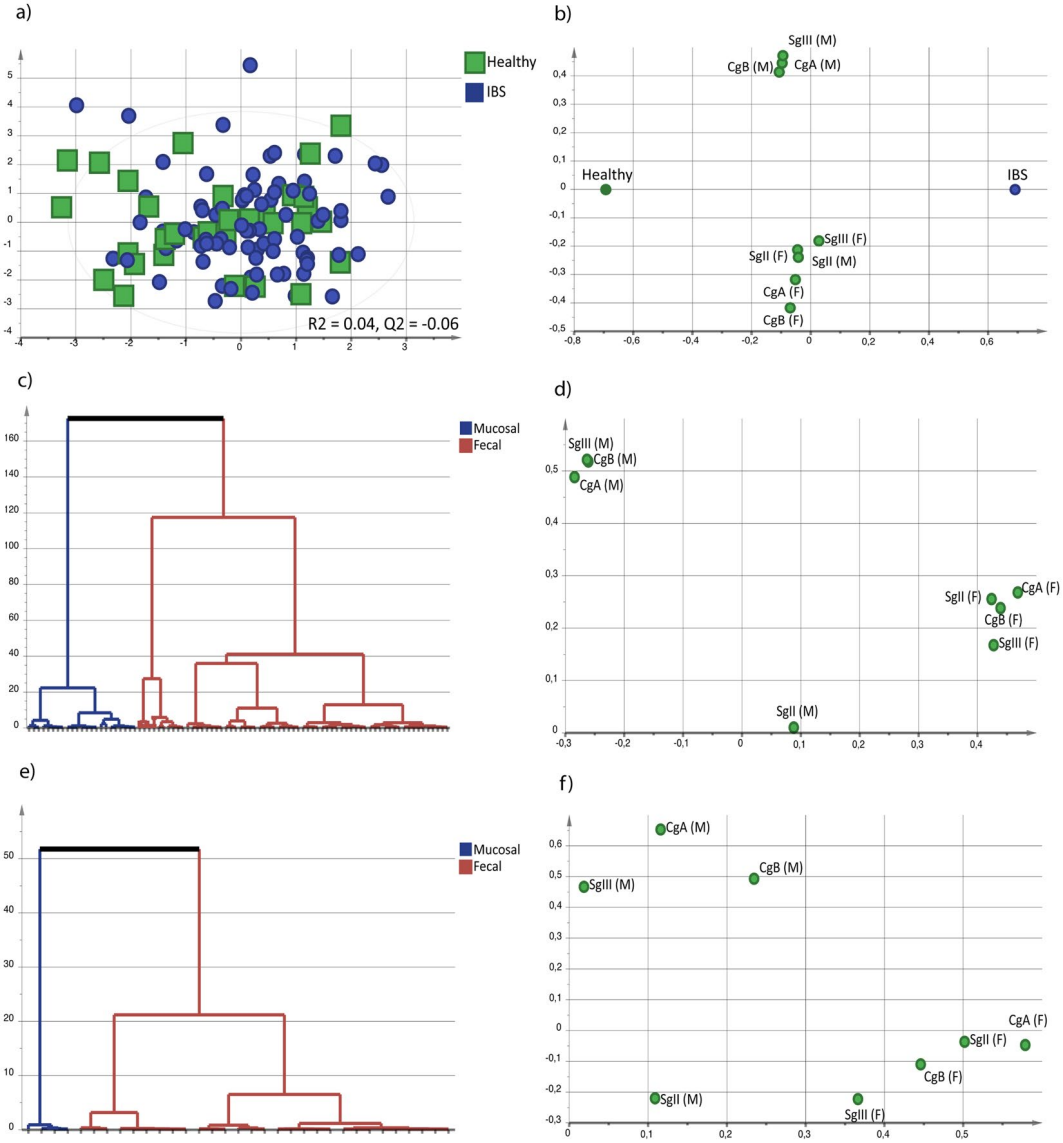
Granin profiles of IBS patients and healthy subjects. Intestinal profiles of fecal protein levels (nmol/L) and mucosal mRNA expression of chromogranins (CgA and CgB) and secretogranins (SgII and SgIII) of irritable bowel syndrome (IBS, n = 88, blue circle) and healthy subjects (n = 33, green square). (**a**) Multivariate orthogonal partial least squares-discriminant analysis (OPLS-DA) scatter plot of IBS patients compared to healthy subjects. (**b**) OPLS-DA loading scatter plot depicting the association between IBS patients and healthy subjects, respectively, and fecal protein levels and mucosal mRNA expression of granins. Multivariate analysis was performed with granins as X-variables and IBS patients and healthy patients, respectively, as Y-variables. (**c**) Dendrogram of the unsupervised bottom-up performed hierarchical clustering analysis (HCA) based on fecal protein levels and mucosal mRNA expression of granins in IBS patients. (**d**) OPLS-DA loading scatter plot depicting the association between granin clusters in IBS (fecal-dominated and mucosal-dominated) and fecal protein levels and mucosal mRNA expression of granins. (**e**) Dendrogram of the unsupervised bottom-up performed hierarchical clustering analysis (HCA) based on fecal protein levels and mucosal mRNA expression of granins in IBS patients. (**f**) OPLS-DA loading scatter plot depicting the association between granin clusters in healthy (fecal-dominated and mucosal-dominated) and fecal protein levels and mucosal mRNA expression of granins.

Further, to determine if intestinal granin profiles of CgA, CgB, SgII and SgIII, based on protein levels in fecal samples and mRNA expression in sigmoid biopsies, differed between IBS patients and healthy subjects, multivariate orthogonal partial least squares-discriminant analysis (OPLS-DA) was performed. IBS patients could not be discriminated (R^2^ = 0.04, Q^2^ = −0.06) from healthy subjects based on the overall profile of fecal and mucosal granins (Fig. 1a). In the loading scatter plot of the OPLS-DA the individual granins were all within close proximity to the centre of the x-axis, confirming low discriminatory power (Fig. 1b).

### IBS patients and healthy subjects display clusters based on fecal and mucosal granins, respectively

Although there were no differences in either protein levels or mucosal expression of granins between IBS patients and healthy subjects, the levels of granins varied substantially within the groups. Therefore, to further explore if these variations were linked to subgroups of IBS patients, clinical characteristics or IBS pathophysiology unsupervised hierarchical clustering analysis (HCA) based on fecal protein levels and mucosal mRNA expression of granins were performed. According to the HCA, IBS patients were subdivided into two clusters: a cluster of subjects characterized by high fecal protein levels of granins (fecal-dominated, n = 65) and a cluster characterized by high mucosal mRNA expression of granins (mucosal-dominated, n = 23, Fig. 1c). The loading scatter plot illustrates the vertical separation between the fecal-dominated granin and mucosal-dominated granin clusters within the IBS patients. The mucosal mRNA expression of granins (CgA, CgB and SgIII) clustered together to the left while the fecal protein levels of CgA, CgB, SgII and SgIII clustered together to the right in the diagram. The mucosal mRNA expression of SgII did not cluster with any other parameter (Fig. 1d).

Similarly to IBS patients, based on fecal protein levels and mRNA expression of mucosal granins, healthy subjects were subdivided into two clusters according to HCA; a fecal-dominated (n = 29) and a mucosal-dominated cluster (n = 4, Fig. 1e). The clustering of fecal and mucosal granins in healthy subjects was similar to the pattern seen in IBS patients, with mucosal mRNA expression of CgA, CgB and SgIII clustering to the left, while fecal protein levels of CgA, CgB, SgII and SgIII cluster to the right (Fig. 1f).

### Fecal and mucosal granins are not related to IBS subtypes or symptom profiles

**Figure 2:**
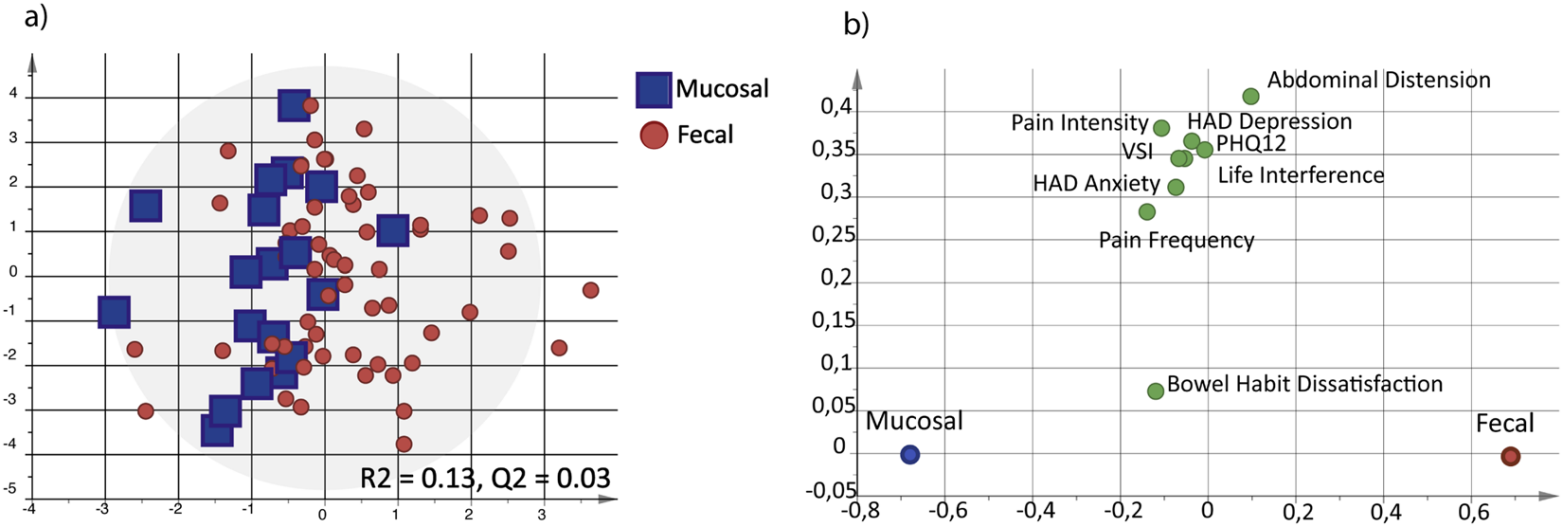
Gastrointestinal and psycological profiles clinical of the fecal-dominated and mucosal-dominated granin clusters of IBS patients. The clinical profiles based on the patients scores on severity of IBS symptoms (IBS-SSS items: pain frequency, pain intensity, abdominal distension, bowel habit dissatisfaction and life interference), non-GI symptoms (PHQ12), anxiety and depression (HADS), and GI-specific anxiety (VSI) were compared between fecal-dominated (red circle, n = 59) and mucosal-dominated (blue square, n = 19) granin clusters of IBS patients (VIP > 0.7). (**a**) Multivariate orthogonal partial least squares-discriminant analysis (OPLS-DA) scatter plot of fecal-dominated and mucosal-dominated granin clusters of IBS patients. (**b**) OPLS-DA loading plot of the clinical symptom profile of the fecal-dominated and mucosal-dominated clusters in IBS patients.

To explore if IBS patients in the fecal-dominated granin cluster had a different gastrointestinal (GI) and psychological symptom profile compared to the IBS patients of the mucosal-dominated granin cluster a multivariate analysis approach was used. There were no differences in symptom profiles between the two clusters (VIP > 0.7, R^2^ = 0.13, Q^2^ = 0.03, Fig. 2a,b), indicating that the different intestinal granin profiles of patients are not linked to IBS symptoms. Further, when comparing IBS subtypes (IBS-D, IBS-C, and IBS-nonCnonD), there were no differences in fecal protein levels or mucosal mRNA expression of CgA, CgB, SgII or SgIII between the IBS subtypes (R^2^ = 0.03, Q^2^ = −0.05). While mucosal CgA mRNA expression was higher in PI-IBS patients compared to non-PI-IBS patients ((0.15 (0.11–0.25) vs. 0.10 (0.06–0.16), *p* < 0.05)), none of the other fecal or mucosal granins differed between these groups.

### No relationship between granins and immune activation in IBS patients

Spearman correlations were performed to explore potential links between granins and the synthesis of serotonin, which is one of the key mediators of gut-brain interactions. In IBS patients mucosal mRNA expression (rho = 0.32, 0.43, 0.23 and 0.33, all *p* < 0.05), but not fecal protein levels of CgA, CgB, SgII and SgIII, was correlated with mucosal tryptophan hydroxylase 1 (TPH1) mRNA expression, suggesting that increased mucosal mRNA expression of granins are linked to increased production of mucosal serotonin. In healthy subjects, none of the fecal or mucosal granins correlated to THP1.

Further, the profiles of immune markers did not differ between the fecal-dominated (n = 58) and mucosal-dominated (n = 19) granin clusters of IBS (VIP > 0.7, R^2^ = 0.26, Q^2^ = −0.07, Supplementary Fig. 1a,b). Although with low predictability, among healthy subjects the fecal-dominated (n = 27) and mucosal-dominated (n = 4) granin clusters showed different profiles of immune markers (VIP > 0.7, R^2^ = 0.65, Q^2^ = 0.25, Supplementary Fig. 1c,d). The complementary loading plot revealed 15 discriminatory immune-related parameters separating the immunological profiles of the fecal-dominated and mucosal-dominated granin clusters of healthy subjects (Supplementary Fig. 1e).

### Granins are associated with the fecal and mucosal microbiota composition

**Figure 3:**
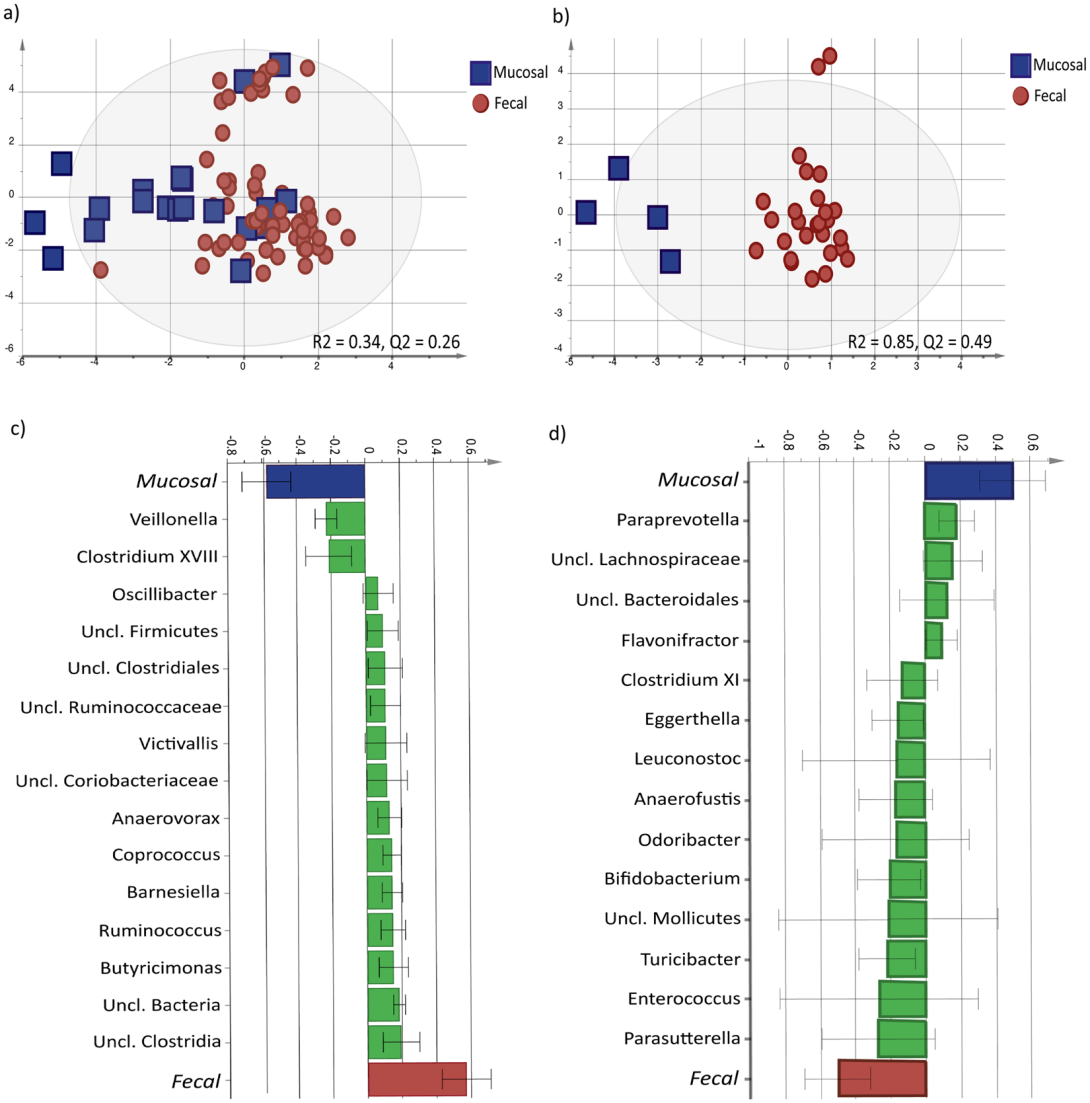
The bacterial composition profiles of IBS patients and healthy subjects based on fecal-dominated and mucosal-dominated granin clusters. The compositions of fecal bacterial genera were compared between of the fecal-dominated and mucosal-dominated clusters of granins in IBS patients and healthy subjects (VIP = 1.35). (**a**) Multivariate orthogonal partial least squares-discriminant analysis (OPLS-DA) scatter plot of the fecal bacterial composition of fecal-dominated (red circle, n = 62) and the mucosal-dominated (blue square, n = 20) clusters of IBS patients. (**b**) Multivariate orthogonal partial least squares-discriminant analysis (OPLS-DA) scatter plot of the fecal bacterial composition of fecal-dominated (red circle, n = 27), and mucosal-dominated granin (blue square, n = 4) clusters of healthy subjects. (**c**) OPLS-DA loadings column plot depicting the fecal bacterial genera that are of most importance for the separation between the fecal-dominated and mucosal-dominated granin clusters of IBS patients. The height of the bar reflects the contribution of each bacterial genus to the separation between the fecal-dominated and mucosal-dominated granin clusters of IBS patients. (**d**) OPLS-DA loadings column plot depicting the fecal bacterial genera that are of most importance for the separation between the fecal-dominated and mucosal-dominated granin clusters of healthy subjects. The height of the bar reflects the contribution of each bacterial genus to the separation between the fecal-dominated and mucosal-dominated granin clusters.

As the next step, we explored if the fecal-dominated and mucosal-dominated granin clusters showed differences in the previously published^23^ fecal and mucosal bacterial profiles in IBS patients and healthy subjects. An OPLS-DA (VIP > 1.35) showed low discrimination between the fecal microbiota composition profiles of the fecal-dominated granin (n = 62) and the mucosal-dominated granin clusters (n = 20) of IBS patients (R^2^ = 0.34, Q^2^ = 0.26) (Fig. 3a). In healthy subjects however, the fecal-dominated granin cluster (n = 27) showed a separate fecal bacterial composition profile (VIP > 1.35) compared to the mucosal-dominated granin cluster (n = 4, Fig. 3b, R^2^ = 0.85, Q^2^ = 0.49). The complementary loading column plot revealed 14 discriminatory fecal bacterial genera separating the fecal bacterial profiles of the fecal-dominated and mucosal-dominated granin clusters of healthy subjects. Of these *Parasutterella*, with a higher relative abundance in the fecal-dominated cluster, and *Paraprevotella*, more abundant in the mucosal-dominated cluster, were the most important genera for the separation between the granin clusters of healthy subjects (Fig. 3c).

**Figure 4:**
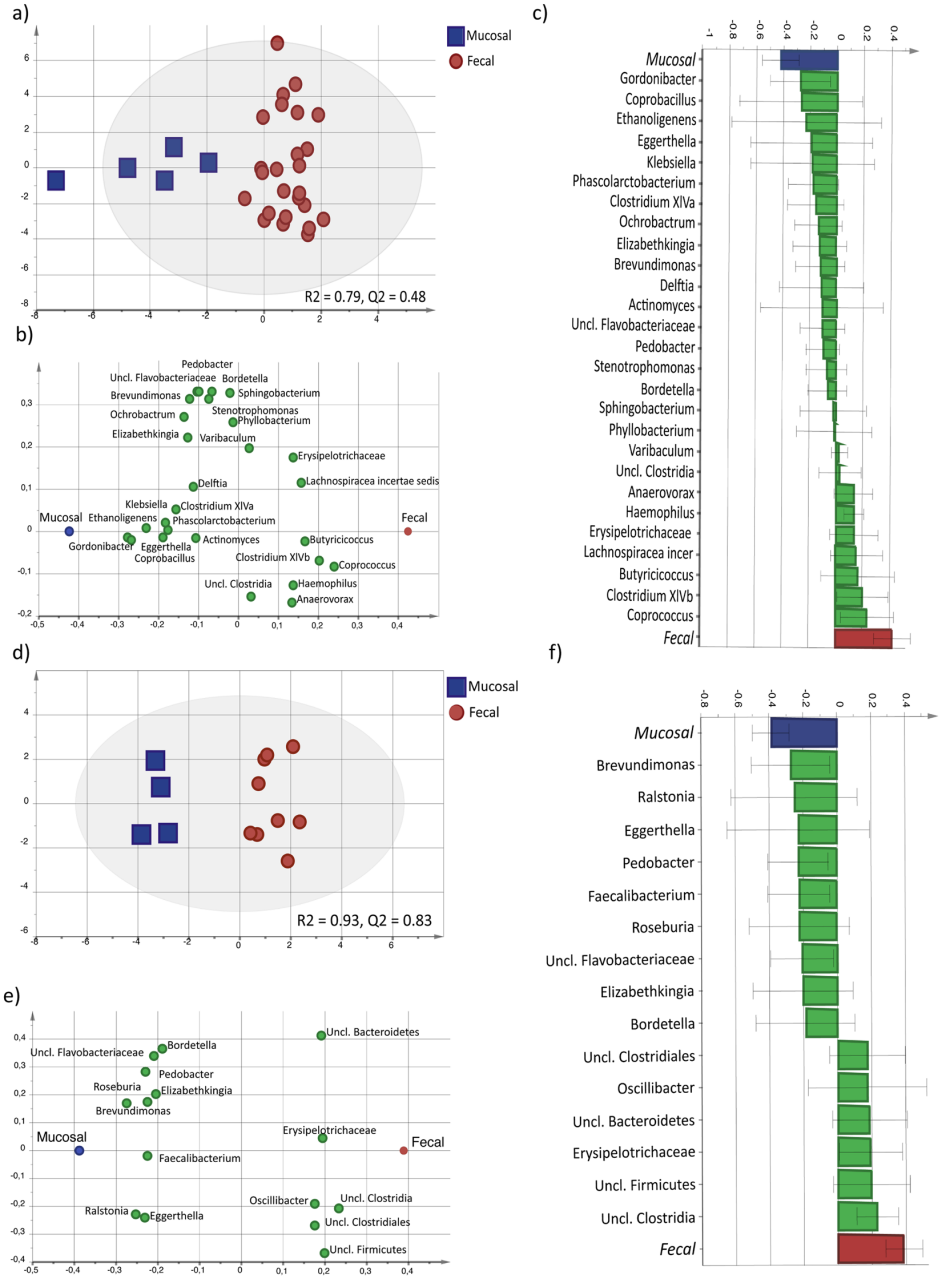
Mucosal bacterial composition profiles of IBS patients clustered into fecal-dominated and mucosal-dominated granin clusters. The composition of the mucosal bacterial genera of fecal-dominated (red circle, IBS n = 25, HS n = 10) and mucosal-dominated clusters of granins (blue square, IBS n = 5, HS n = 4) was compared between IBS patients and healthy subjects (VIP = 1.35). (**a**) OPLS-DA scatter plot of the mucosal bacterial composition of fecal-dominated (red circle, n = 25) and mucosal-dominated (blue square, n = 5) granin clusters of IBS patients. (**b**) OPLS-DA loading plot of the discriminatory bacterial genera separating the mucosal bacterial composition profile of the fecal-dominated (right) and mucosal-dominated (left) clusters of IBS. (**c**) OPLS-DA loadings column plot depicting the mucosal bacterial genera that are of most importance for the model of the mucosal bacterial profile of fecal-dominated and mucosal-dominated granin clusters of IBS. The height of the bar reflects each bacterial genus contribution to the separation between the fecal-dominated and mucosal-dominated granin clusters. (**d**) OPLS-DA scatter plot of the mucosal bacterial composition of fecal-dominated (red circle, n = 10) and mucosal-dominated (blue square, n = 4) granin clusters of healthy subjects). (**e**) OPLS-DA loading plot of the discriminatory bacterial genera separating the mucosal bacterial composition profile of fecal-dominated (right) and mucosal-dominated (left) granin clusters of healthy subjects. (**f**) OPLS-DA loadings column plot depicting the mucosal bacterial genera that are of most importance for the model of fecal-dominated and mucosal-dominated granin of healthy subjects. The height of the bar reflects each bacterial genus contribution to the separation between the fecal-dominated and mucosal-dominated granin clusters.

Furthermore, the mucosal bacterial profiles of the fecal-dominated and mucosal-dominated granin clusters of IBS patients (n = 30) and healthy subjects (n = 14) were determined. Among IBS patients, the fecal-dominated granins cluster (n = 25) displayed a separate mucosal microbiota profile compared to the mucosal-dominated granin cluster (n = 5) (VIP > 1.35, R^2^ = 0.79, Q^2^ = 0.48) (Fig. 4a,b). Twenty-seven bacterial genera separated the clusters, of which the *Gordonibacter* was more abundant in the fecal-dominated granin cluster, and *Coprococcus* more abundant in the mucosal-dominated granin cluster (Fig. 4c). Also in healthy subjects, the mucosal bacterial profile of the fecal-dominated granin cluster (n = 10) differed compared to the mucosal-dominated granin cluster (n = 4) (VIP > 1.35, R^2^ = 0.93, Q^2^ = 0.83) (Fig. 4d,e). Sixteen bacterial genera were driving the separation in mucosal bacterial composition between the fecal-dominated and mucosal-dominated granin clusters, of which undefined Clostridia, more abundant in the fecal-dominated granin cluster, and *Brevundimonas*, more abundant in the mucosal-dominated granin cluster, were the most important (Fig. 4f). In addition, several discriminatory bacterial genera such as *Pedobacter*, *Unspecified Flavobacteriaceae*, *Eggerthella*, *Elisabethkingia* and *Bordetella* of the mucosal microbiota were of higher relative abundance in the mucosal-dominated granin cluster of both IBS patients and healthy subjects, while mucosal *Clostridia* were of higher relative abundance in the fecal-dominated granin cluster of both IBS patients and healthy subjects (Fig. 4c,f).

### Higher fecal protein levels of granins are associated with a less diverse bacterial composition and the Bacteroides enterotype

Further, we explored if granins are linked to bacterial alpha diversity (richness as measured by number of OTUs). In IBS patients, fecal alpha diversity was negatively correlated with fecal protein levels of CgA, CgB, SgII and SgIII (Table 2). Similarly, in healthy subjects fecal bacterial alpha diversity was negatively correlated with SgIII (Table 2). In contrast, there were no associations between mucosal mRNA expression of any of the chromogranins or secretogranins and bacterial alpha diversity in either fecal or mucosal samples of IBS patients or healthy subject (Table 2).

**Table 2:** Correlations (rho) between fecal protein levels and mucosal mRNA expression of chromogranins (CgA, CgB) and secretogranins (SgII, SgIII) and fecal bacterial Richness (alpha-diversity).

(IBS = 131)		Fecal (n = 82)	Mucosal (n = 30)	(HS = 31)		Fecal (n = 82)	Mucosal (n = 30)
		Alpha diversity	Alpha diversity			Alpha diversity	Alpha diversity
Fecal	CgA	−0.28**	−0.19	Fecal	CgA	−0.33	−0.30
	CgB	−0.31**	−0.15		CgB	−0.05	−0.34
	SgII	−0.29**	−0.16		SgII	**−0.35**	−0.25
	SgIII	−0.34**	−0.19		SgIII	−0.39*	−0.50**
Mucosal	CgA	−0.12	0.06	Mucosal	CgA	0.11	0.13
	CgB	−0.05	−0.01		CgB	0.01	−0.23
	SgII	−0.21	−0.18		SgII	0.01	−0.36
	SgIII	−0.01	0.21		SgIII	0.09	0.10

Abbreviations: CgA = Chromogranin A, CgB = Chromogranin B, SgII = Secretogranin II, SgIII = Secretogranin III, Spearman correlations, *p* < 0.05. **p < 0.01. ***p < 0.005.

**Figure 5:**
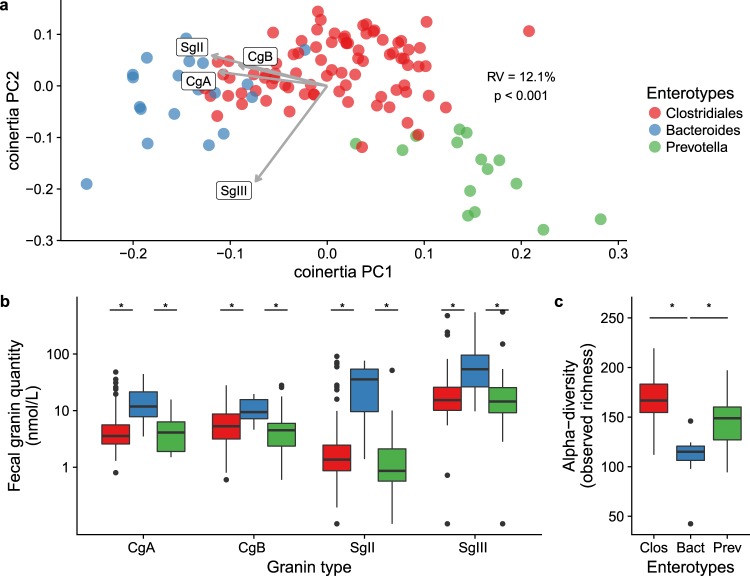
The association between fecal granin levels with fecal bacterial diversity and enterotypes of IBS patients (n = 86) and healthy subjects (n = 32). (**a**) Principal coordinate plot of co-inertia analysis between fecal granins (CgA, CgB, SgII and SgIII) with bacterial beta diversity in faeces of IBS patients and healthy subjects (JSD distance) coloured according to enterotype (*Bacteroides* n = 19, blue, *Clostridiales* n = 84, red and *Prevotella* n = 15, green). Each dot represents one individual. Grey arrows indicate the association direction and size of CgA, CgB, SgII and SgIII, respectively. (**b**) Comparison of fecal protein levels of granins (CgA, CgB, SgII and SgIII) of IBS patients and healthy subjects subdivided according to their enterotypes. (**c**) Comparison of the bacterial alpha diversity in faeces between the *Bacteroides*, *Clostridiales* and *Prevotella* enterotypes of IBS patients and healthy subjects.

Finally, to investigate the link between fecal protein levels of granins and the bacterial composition of IBS patients and healthy subjects, participants were subdivided into enterotypes based on their fecal microbiota. Co-inertia analysis of IBS patients and healthy subjects based on beta-diversity (JSD) of microbiota composition, revealed separate clusters of the bacterial enterotypes and showed a negative association between the abundance of all fecal granins (CgA, CgB, SgII and SgIII) with SgII as the most important factor contributing to the variation along the first co-inertia component (PC1), (RV = 0.12, p < 0.001; Fig. 5a). Further, in IBS patients and healthy subjects the *Bacteroides* enterotype (n = 15) had higher fecal protein levels of granins (CgA, CgB, SgII and SgIII) but lower bacterial alpha diversity compared to the *Clostridiales* (n = 59) and *Prevotella* enterotype (n = 8, Fig. 5b,c).

## Discussion

This exploratory study demonstrated a low discrimination between IBS patients and healthy subjects based on the intestinal granin profile. However, two distinct clusters dominated by high levels of fecal granins or high expression of mucosal granins were identified both in IBS patients and healthy subjects, and the fecal-dominated and mucosal-dominated granin clusters of IBS patients and healthy subjects demonstrated separate fecal and mucosal bacterial profiles. Additionally, bacterial diversity and enterotypes were strongly linked to the intestinal granin profiles.

In the this study, we identified a high inter-individual variability of the fecal protein levels and mucosal mRNA expression of chromogranins and secretogranins, revealing a cluster with high protein levels of granins in faeces, and another cluster with high mucosal mRNA expression of granins, within both the IBS and healthy populations. As IBS consists of patients with not only heterogeneous symptom profiles but also a complex and heterogeneous pathophysiology, e.g. in terms of immune activation and bacterial dysbiosis^2,5,18,23^, further evaluation of links between these granin clusters with IBS symptoms and pathophysiology were investigated. Based on these clusters, we demonstrated that fecal protein levels and mucosal expression of granins are strongly associated with the mucosal and fecal bacterial composition in both IBS patients and healthy subjects. While many bacteria that were associated with either the fecal or mucosal granin clusters respectively, overlapped between IBS patients and healthy subjects, other bacteria only showed association to granins in either of the groups. This could hypothetically reflect divergences in the bacterial composition of the IBS patients in this study cohort^23^ or be a result of aberrant mucosal responses to bacteria as previously shown *ex vivo in* IBS patients compared to healthy subjects^24^. The underlying mechanism for the strong association between the mucosal mRNA expression of granins and the mucosal adherent bacterial composition remains to be determined. However, it may be suggested that granins expressed in the sigmoid colon have strong local influence on the adherent mucosal bacterial composition. On the other hand, fecal protein levels of granins are the net result of the secretion of granins in the entire intestine and might therefore correlate less well with adherent mucosal microbiota composition of the sigmoid colon. Further, among the mucosa adherent microbiota the discriminatory bacterial genera were similar in IBS patients and healthy subjects, which suggest that fecal protein levels and mucosal expression of granins, respectively, may influence certain bacterial genera rather than having a general inhibitory effect on bacterial growth.

Similarly, levels of fecal granins located in the lumen were strongly associated with fecal microbiota composition, whereas the link to mucosa-associated microbiota was weaker. For instance, independent of granin type, there was a negative relationship between fecal protein levels of CgA, CgB, SgII and SgIII and bacterial diversity in faeces, but not mucosa, of IBS patients, and a similar pattern was seen among healthy subjects. Even though we in this study could not confirm previous findings of an association between CgA with IBS, our findings demonstrating a negative association between fecal protein levels of CgA and fecal bacterial diversity, are in line with a previous large Dutch population-based study^25^. Notably, in addition to CgA this study shows that also fecal protein levels of other granins CgB, SgII and SgIII are associated with a less diverse bacterial composition. Particularly, SgII seems to be a strong marker of a less diverse bacterial composition.

In addition, we have demonstrated that individuals with the *Bacteroides* enriched enterotype, previously shown to be associated with low bacterial diversity^23,26^, display higher protein levels of CgA, CgB, SgII and SgIII in faeces. Enterotypes have been proposed to classify individuals according to their microbiota configuration, and have been shown to be closely associated with long-term diet^27^, gut transit time^23^, immune senescence and low-grade inflammation^26^, as well as IBS symptom severity^23^. Our findings therefore suggest that fecal protein levels of granins may be linked to diet-associated host regulation of the bacterial composition, potentially to maximize the digestion of nutrients, which in turn could influence gut health. Thus, fecal granins might be a host response directed to shape the intestinal bacterial composition, potentially to limit the growth of pathogens or opportunistic bacteria or to engineer the composition to allow maximal metabolic effect.

Several potential direct and indirect mechanisms for how granins regulate the intestinal bacterial composition have been demonstrated by other studies *in vitro*. For instance, granins have been shown to reduce luminal bacterial growth by their acidity and granin-derived peptides exerts bacteriolytic effects^28,29^. However, a previous study that measured the isoelectrical point (IEP)) of the granins showed that CgB had the highest IEP of the granins^30^, while in this study, CgB showed slightly lower correlation to bacterial alpha diversity compared to SgII with lower IEP. These findings support the notion that granins may have a regulatory effect on the bacterial composition beyond their acidity. Apart from having a direct effect on the bacterial composition, granins may potentially also have immune mediated influences on gut microbiota composition. Granins showed stronger association to both fecal and mucosal microbiota composition in healthy subjects than in IBS patients, and a link between granins and immune activation was only identified in healthy subjects. This lack of association between immune activation and granins in IBS patients requires further elucidation, but one may speculate in that granins may be involved in the aberrant mucosal immune response to bacteria, previously shown in *in vitro* mucosal cultures of IBS biopsies^31^. However, we cannot rule out that the lack of association between immune activation and granins are a result of an unknown direct influence of granins on bacterial growth.

The strong association between fecal granin levels and the bacterial composition, which in animal models has been shown to have a vital role in the gut-brain interaction affecting brain function and behaviour^32^ suggests that granins may have a central role in the communication between the intestine and the central nervous system (CNS). Further support in our study for the role of granins in the regulation of the gut-brain interaction is the moderate correlation between mucosal mRNA levels of granins with the rate-limiting enzyme in serotonin synthesis, THP1, in IBS patients. This finding suggests that granin production is linked to serotonin, which in turn affect gut motility, pain perception and facilitate gut-brain communication^33^. Hence, mucosal granin expression could have an effect on gut transit, visceral hypersensitivity and CNS function. However, neither fecal protein levels nor mucosal mRNA expression of granins were associated with psychological or gastrointestinal symptoms in IBS patients.

This descriptive cross-sectional study, allows for identification of relationships between intestinal granins and fecal and mucosal bacterial genera, and suggest that granin analysis potentially could be used as relatively inexpensive and clinically accessible biomarker for intestinal bacterial dysbiosis. However, this study does not provide knowledge of how granins interact or regulate the growth of bacterial genera. Further, as the majority of the symptoms of the patients originate from the intestine and granins have been suggested to have several local effects in the GI tract on the mucosal immune system, barrier integrity and, at least in animal models, bacterial composition and gut motility^21,22,34,35^ the focus of this study has been on intestinal granins. However, we do not exclude the possibility that serum chromogranins are a useful biomarker of IBS, as has previously been suggested for IBD^36^. One of the limitations of this study is that among the healthy subjects only four individuals belonged to the cluster characterized by high mucosal expression levels of granins. Therefore, the association between this cluster and immunological and microbial parameters should be interpreted with caution. Further, this study cannot rule out the possibility that the relationship between fecal protein levels and mucosal mRNA expression of chromogranins and secretogranins reflect the host’s response to bacterial dysbiosis, aiming to restore the intestinal bacterial balance. The origin of fecal granins has not been evaluated in this study and therefore future mechanistic studies of intestinal cells and their granin secretion under different physiological conditions are needed. As compared to a previous study exploring the link between fecal protein levels of CgA and bacterial dysbiosis^25^, our study however also investigated fecal protein levels of CgB, SgII and SgIII as well as mucosal mRNA expression of granins and demonstrated that these less explored granins potentially have a strong role in the regulation of the gut flora. Further, the extensive, clinical, bacterial and immunological characterization of IBS patients and healthy controls in this study enabled us to explore linkages between granins and the IBS symptom profile, immune activation and the link to serotonin synthesis, which previously has not been investigated.

To conclude, this study demonstrated that fecal protein levels and mucosal mRNA expression of CgA, CgB, SgII and SgIII are linked to the gut bacterial composition, bacterial diversity and enterotypes in IBS patients and healthy subjects, without any major differences between patients and controls, and with no clear association with symptoms. Taken together with previously shown effects on bacterial growth^28^, these findings suggest that granins may be one of several host-produced factors being part of the microbiota-gut-brain interaction and regulating the microbiota composition of the intestine, potentially limiting the growth of opportunistic bacteria or engineering the bacterial composition to allow maximal metabolic effect.

## Method

### Study population and material sampling

IBS patients who met the ROME III diagnostic criteria^37^ were recruited from the outpatient clinic at Sahlgrenska University Hospital Gothenburg, Sweden. Routine histology defined biopsies of IBS patients as non-inflammatory and exclusion criteria included presence of Crohn’s disease, Ulcerative colitis, collagenous and lymphocytic colitis based on standard criteria^38^, celiac disease based on clinical history and negative tissue transglutaminase (tTg)-IgA antibodies, food allergies, or any other GI disease explaining the symptoms. Twelve IBS patients were on stable doses of anti-depressants, mainly selective serotonin reuptake inhibitors (SSRIs). Other exclusion criteria included abnormal results on standard screening laboratory tests, severe psychiatric, systemic or other chronic diseases, history of drug or alcohol abuse, and the inability to reliably respond to questionnaires in Swedish. Patients who described a sudden onset of their IBS symptoms following presumed gastroenteritis, although not confirmed through bacterial stool culture, were regarded as having post-infectious IBS (PI-IBS).

Based on recorded bowel movements in the two-week stool diary Bristol stool form (BSF), IBS patients were subtyped into diarrhoea-predominant IBS (IBS-D) or constipation-predominant IBS (IBS-C), while IBS patients with mixed loose and hard stools (IBS-M) and those who had unsubtyped IBS (IBS-U) were combined into one group (IBS-nonCnonD)^37^. Patients with incomplete BSF diaries were subtyped at the outpatient clinic based on clinical history by the treating physician. Healthy subjects without current gastrointestinal (GI) symptoms (assessed seven days prior to inclusion), psychiatric, gastrointestinal, cardiac or metabolic diseases were recruited through advertisement. IBS patients and healthy subjects were of Caucasian ethnic origin. Informed consent was obtained from all participants

From all study subjects, sigmoid colonic biopsies (25–35 cm proximal from the anus) were obtained using standard biopsy forceps without prior bowel preparation. Once collected, biopsies for expression analysis were stored in RNA-later (Ambion, Austin, TX, USA) overnight, while biopsies for microbiota analysis were immediately frozen in liquid nitrogen. All biopsies were stored at −80 °C until further analysis. For the extraction of serum, venous blood samples were collected without additives and centrifuged at room temperature and the supernatant was frozen in −80 °C until analysis. Fecal samples for protein analysis of granins were collected by patients at home, immediately frozen in −20 °C and brought frozen to the laboratory while fecal samples for microbiota analysis were collected by patients at home in RNA-later and stored at room temperature up to three weeks. All fecal samples were stored frozen in −80 °C until analysis.

The study was approved by the Regional Ethical review Board in Gothenburg. All methods in this study were performed in accordance with the relevant guidelines and regulations.

### Analysis of granins and calprotectin in fecal samples

Protein extraction from fecal samples was performed with one part faeces and 49 parts of extraction buffer, as previously described^7^. Fecal protein levels of CgA and CgB were measured with radioimmunoassay (Eurodiagnostica, Malmö, Sweden), according to the manufacturer’s instructions. Fecal protein levels of SgII and SgIII were measured with in-house radioimmunoassay at Uppsala University^39^. In brief, samples were diluted in assay buffer (0.05 M Na_3_PO_4_ buffer, pH 7.4, with 0.15 M NaCl, 0.02% NaN_3_, 0.2% BSA and 0.5% Tween 20). The peptides were labelled with ^125^I (MP Biomedicals, Doornveld, Belgium) using the chloramine-T method as previously described^40^. Standards and samples, in duplicates, were incubated for 3 days at 4 °C with tracer (30,000 c.p.m/tube) and primary antibodies that were diluted to give 30% bound radioactivity. Free tracer was separated by secondary antibody (goat anti-rabbit IgG coupled to solid phase, SAC Cel Anti-rabbit, IDS Nordic, Herlev, Denmark). Antibody-bound radioactivity was measured in a γ counter (Auto gamma, Wallac, Pharmacia Biotec, Uppsala, Sweden). Data calculation was performed with a legit-log transformation program (multiclass, Wallac, Pharmacia Biotec, Uppsala, Sweden). Fecal calprotectin was analysed with commercial ELISA accordingly to the manufacturer’s instructions (Buhlmann Calprotectin ELISA kit, Buhlmann Laboratories, Shönenbuch, Switzerland).

### Mucosal biopsy expression analysis of immune parameters

Mucosal expression of pro- and anti-inflammatory cytokines and markers of permeability were investigated in the subjects. Quantitative reverse transcription PCR (RT-qPCR) analysis to quantify the mRNA expression of CgA, CgB, SgIII, interleukin (IL)-8, IL-10, tumour necrosis factor (TNF), forkhead box P3 (FoxP3), occludin (OCLN) and zonulin-1 (ZO-1), Nikotinamid-adenin-dinukleotidfosfat oxidase 1 (NOX1), toll-like receptor (TLR2), TLR6 and TLR9 and tryptophan hydroxylase 1 (TPH1) was conducted in mucosal biopsies with a NucleoSpin® RNA Kit, following manufacturers’ protocol (Ref. 740955.50, Macherey-Nagel, Düren, Germany). In brief, biopsies were placed in lysis buffer and homogenized for 2 × 2 min. Extracted m-RNA was stored at −80 °C until the cDNA was transcribed (Master mix Reverse Transcription Kit, Ref. 11755250, Life Technologies) and transferred to 96 well plates. Polymerization was performed by ≪Taqman® Fast Advanced Master Mix≫ (Ref. 444965, Life Technologies). The average expression of reference genes 18S, GAPDH and HPRT (CgA, CgB, SgIII, and toll-like receptor (TLR2), TLR6 and TLR9) or 18S, POLR2YA and RPLP0 (SgII, TNF, OCLN, TPH1, FoxP3, zonulin-1 (ZO-1), interleukin (IL)-8, IL-10,) was used to normalize the expression of the targeted genes. The difference between the average expression of reference genes and that of the sequence of interest is given as ∆Ct (cycle threshold) and presented as 2^−(Target-Reference)^ or (2^∆Ct^).

### Serum analysis of cytokines and permeability markers

Serum levels of pro and anti-inflammatory immune markers have previously been investigated in the subjects included in this study. In brief, measurement of interleukin (IL)-5, IL-6, IL-8, IL-10, IL-12p70, IL-13, IL-17A, interferon gamma (IFN-γ), lipopolysaccharide binding protein (LBP) and tumour necrosis factor (TNF) was carried out using Meso Scale Discovery (MSD) multiplex immune assay according to manufacturer’s instructions (MSD SCALE DISCOVERY, Rockville, USA) as previously described^2^. Zonulin were analysed with commercial ELISA accordingly to the manufacturer’s instructions (Nordicbiosite, Täby, Sweden).

### Microbiota composition assessment in fecal and mucosal samples

DNA was extracted as previously described from faeces (n = 113) and biopsies (n = 44)^41^. In short, hypervariable 16S rRNA regions (V5-V6) were amplified using primers 5′-AGGATTAGATACCCTGGTA-3′ and 5′-CRRCACGAGCTGACGAC-3′. Sequencing was done by DNA Vision SA (Charleroi, Belgium) on a 454 Life Sciences Genome Sequencer FLX instrument (Roche) using titanium chemistry. Raw reads quality filtering and trimming, OTU (operational taxonomic units) clustering, taxonomic assignment were performed using the LotuS v1.32 pipeline28^42^. The relative abundance of 188 bacterial genera was used for analysis. α-diversity was evaluated on Shannon index and number of OTUs following rarefaction at 1100 sequences using the vegan R package. Beta diversity (diversity between samples) was performed on Jensen-Shannon distance on genus level^23^. Enterotype stratification was identified in fecal samples using previously described methods with the Dirichlet multinomial mixture model^43^.

### Questionnaires

Demographic and disease-related data were collected during structured interviews and patients completed the following self-administered questionnaires;

#### The IBS Severity Scoring System (IBS-SSS)

IBS-SSS, which includes five items—pain severity, pain frequency, severity of abdominal distension, bowel habit dissatisfaction and daily life interference, was used to assess IBS symptom severity. The scores are combined into an overall IBS score ranging from 0 (no symptoms) to 500 (maximum severity)^44^. We used the total score of this questionnaire to analyse the severity of IBS.

#### Visceral sensitivity index (VSI)

The VSI was used to assess GI-specific anxiety, i.e. the cognitive, affective, and behavioural responses to the fear of GI sensations and symptoms and in which context these occur. The instrument contains 15 questions, each with a 6-grade scale (strongly agree-strongly disagree), with higher scores meaning more severe GI-specific anxiety^45^.

#### Hospital anxiety and depression (HAD)

To detect severity of anxiety and depression, IBS patients and healthy subjects answered 14 questions using a four point Likert scale ranging from 0–3. For each subscale, seven questions relate to anxiety and seven to depression with a minimum and maximum score of zero and three respectively with a high score indicating more severe symptoms^46^.

#### IBS-quality of life (QoL)

The IBSQoL is a disease-specific questionnaire used to evaluate emotional functioning, mental health, physical functioning, energy, sleep, food/diet, social role, physical role, and sexual relations in IBS patients. To facilitate score interpretation, the summed total score is transformed to a 0–100 scale ranging from 0 (poor QoL) to 100 (maximum QoL).

#### The Patient Health Questionnaire (PHQ)-12

The Patient Health Questionnaire (PHQ)- 15 asks the subject about the severity of 15 somatic symptoms, each scored from 0 (not bothered at all) to 2 (bothered a lot)^47^. In this study the three GI symptoms were excluded and instead PHQ-12 was used, to have a measure specifically for non-GI symptoms^48^.

### Data processing and statistical analysis

#### Multivariate analysis

Multivariate factor analyses were applied to investigate if fecal protein levels or mucosal mRNA expression of granins (CgA, CgB, SgII and SgIII), fecal and mucosal bacterial genera, immune factors as well as clinical measurements (X variables) discriminated between groups of study subject (Y variables).

Variables included in multivariate analysis were transformed if their distributions were skewed. Subjects with more than 60% missing variables and variables with only one observation different from the mean were defined as outliers and excluded from multivariate analysis. In multivariate analysis outliers were removed if they were both above Hotelling’s T2 Range Line of 95% and DModX DCrit (0.05).

Orthogonal partial least squares-discriminant analysis (OPLS-DA) was performed to discriminate between IBS patients and healthy subjects and clusters thereof (Version 14.1.3.0, copyright © MKS Data Analytics Solutions). The R^2^ parameter represents the fit of the OPLS-DA with the best possible fit being R^2^ = 1, while when considering heterogeneous biological variables, a model would be considered to have a good fit with an R^2^ ≥ 0.5. The Q^2^ parameter represents the predictive ability calculated by cross-validation, with the best value of Q^2^ = 1, while a Q^2^ value > 0.4 is considered good with biological variables^49^. The loading scatter plot may be superimposed over the OPLS-DA plot to see the associations between X-variables and individuals. Loading plots were generated to identify the X-variables most important for the discrimination between Y-variables (clusters). Parameters localized further away from the centre of the x-axis contribute more to the discrimination of the clusters in the loading scatter plot. In the loading column plot, variables with a large impact and more reliable contribution have larger columns and smaller confidence intervals. For immune and clinical parameters Variable Importance for the Projection (VIP) >0.7 was used. However, when modelling the fecal and mucosal microbiota, the number of bacterial genera exceeded the number of subjects by far. Therefore, bacterial genera that contributed most to the underlying variation in the X variables (bacterial genera) were identified with a VIP > 1.35. To reduce the risk of over-fit, post-hoc 100 permutation tests of OPLS-DA models were performed and only models with R^2^ < 0.4 and Q^2^ < 0.05 were accepted^50^. Unsupervised hierarchical cluster analysis (HCA) with distance calculated by ward was used to identify the fecal-dominated and mucosal-dominated clusters of IBS patients and healthy controls, based on their fecal protein levels and mucosal mRNA expression of granins. The heights of the clusters are proportional to the distance between clusters.

As previously described in Tap *et al*.^23^, co-inertia analysis is an ordination method for coupling 2 (or more) sets of parameters (e.g., abundance of fecal granins (CgA, CgB, SgII and SgIII) and microbiota OTU proportion) by looking at their linear combinations. In the co-inertia analysis, the co-inertia (the sum of the square of covariance) between the 2 sets is maximized and decomposed. Hence, the co-inertia value, so called RV coefficient, is a global measure of the co-structure between the 2 data sets^51^. The co-inertia RV coefficient is high when the 2 sets vary dependently and low when they vary independently. Co-inertia analysis was performed on fecal microbiota beta diversity and the abundance of fecal granins (CgA, CgB, SgII and SgIII). In this study, we used co-inertia analysis based on fecal microbiota composition using JSD distance metric and successively coupled with a principal component analysis computed fecal levels of granins. A Monte Carlo test was used to test the robustness of the RV coefficient.

#### Univariate analysis

Univariate analysis was performed in GraphPad Prism 6 for Mac (GraphPad Software, La Jolla, CA). Mann-Whitney was used to assess differences between two clusters and Kruskal-Wallis followed by Dunn’s test, was implemented for differences between three or more groups. Non-parametric Spearman’s rank coefficient (rho) was used for analyses of correlations. *p* values < 0.05 were considered as statistically significant. If no other specification, results are presented as median ± (25–75% percentile).

## Electronic supplementary material

Supplementary Information
