# Supplementary material for: Fecal chromogranins and secretogranins are linked to the fecal and mucosal intestinal bacterial composition of IBS patients and healthy subjects
Source: Sci Rep. 2018 Nov 14;8:16821. doi: 10.1038/s41598-018-35241-6 (PMC6235916; doi:10.1038/s41598-018-35241-6)
Supplement: Supplementary file 1 — Supplementary Information [file 41598_2018_35241_MOESM1_ESM.pdf]

Fecal chromogranins and secretogranins are linked to the fecal and mucosal intestinal bacterial composition of IBS patients and healthy subjects

Short running title: Granins are linked to the bacterial composition

Johanna Sundin[1,2], Mats Stridsberg[3], Julien Tap[4], Muriel Derrien[4], Boris Le Nevé[4], Joel Doré[5], Hans Törnblom[1], **Magnus Simrén[1,6*], Lena Öhman[1,2*].**

* Shared senior authorship

[1]Inst. of Medicine, University of Gothenburg, Sweden [2]Inst. of Biomedicine, University of Gothenburg, Sweden [3] Department of Medical Sciences, Uppsala University, Uppsala; Sweden [4]Danone Nutricia Research, Department of Innovation, Science and Nutrition, Palaiseau, France. [5]French National Institute for Agricultural Research (INRA) MetaGenoPolis, France. [6] Centre for Functional Gastrointestinal and Motility Disorders, University of North Carolina at Chapel Hill, Chapel Hill, NC, USA.

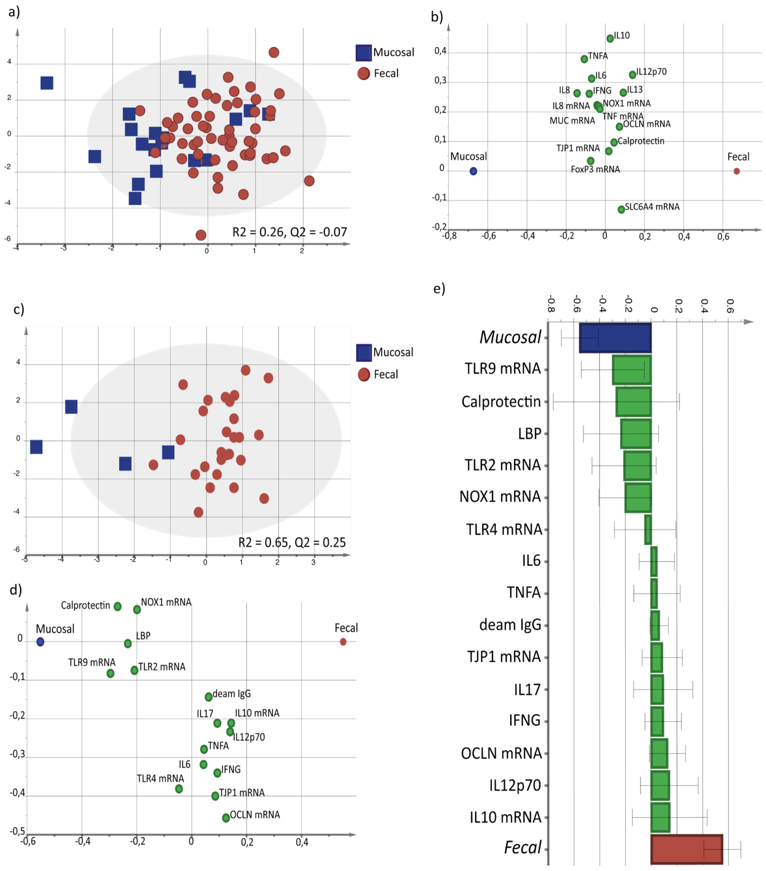

a)
Mucosal
Fecal
R2 = 0.26, Q2 = -0.07
b)
IL10
TNFA
IL12p70
IL6
IL8
IFNG
IL13
IL8 mRNA
NOX1 mRNA
MUC mRNA
TNF mRNA
OCLN mRNA
Calprotectin
TJP1 mRNA
FoxP3 mRNA
SLC6A4 mRNA
Mucosal
Fecal
c)
Mucosal
Fecal
R2 = 0.65, Q2 = 0.25
d)
Calprotectin
NOX1 mRNA
Mucosal
LBP
Fecal
TLR9 mRNA
TLR2 mRNA
deam IgG
IL17
IL10 mRNA
IL12p70
TNFA
IL6
IFNG
TLR4 mRNA
TJP1 mRNA
OCLN mRNA
e)
Mucosal
TLR9 mRNA
Calprotectin
LBP
TLR2 mRNA
NOX1 mRNA
TLR4 mRNA
IL6
TNFA
deam IgG
TJP1 mRNA
IL17
IFNG
OCLN mRNA
IL12p70
IL10 mRNA
Fecal

Supplementary figure 1. ***Global immune- and permeability profile of IBS patients and healthy subjects based on fecal-dominated and mucosal-dominated granin clusters.*** *The global immune- and permeability profile of fecal-dominated (red circles, IBS n = 58, HS n = 27) and mucosal-dominated (blue squares, IBS n = 19, HS n = 4) granin clusters of IBS patients and healthy subjects (VIP > 0.70). a) Multivariate orthogonal partial least squares-discriminant analysis (OPLS-DA) scatter plot showing low discrimination in the global immune- and permeability profile of fecal-dominated (red circles, n = 58) and mucosal-dominated (blue squares, n = 19) granin clusters of IBS patients. b) OPLS-DA loading plot of the discriminatory bacterial genera separating the global immune- and permeability profile of fecal-dominated and mucosal-dominated granin clusters of IBS patients. c) OPLS-DA scatter plot showing discrimination in the global immune- and permeability profile of fecal-dominated and mucosal-dominated granin clusters of healthy subjects. d) OPLS-DA loading plot of the discriminatory immunological factors separating the immune profile of fecal-dominated (right) and mucosal-dominated granin (left) clusters of healthy subjects. e) OPLS-DA loadings column plot depicting the immunological factors that are of most importance for the model of fecal-dominated (red circles, n = 27) and mucosal-dominated (blue squares, n = 4) granin of healthy subjects. The height of the bar reflects the contribution of each immunological factor to the separation between the fecal-dominated and mucosal-dominated granin clusters of healthy.*

Supplementary table 1. Fecal levels and mucosal expression of chromogranins (CgA. CgB) and secretogranins (SgII. SgIII) in irritable bowel syndrome patients (IBS, n = 88) and healthy subjects (HS, n = 33)

		CgA	*p*-value	CgB	*p*-value	SgII	*p*-value	SgIII	*p*-value
Fecal (nmol/L)	**IBS**	3.9 (2.5-7.9)	0.52	5.7 (3.2-8.8)	0.69	1.3 (0.8-6.8)	0.43	18.6 (12.4-38.0)	0.20
	**HS**	4.7 (2.9-9.0)		5.6 (3.1-10-0)		1.9 (0.9-3.6)		14.4 (8.7-48.2)	
Mucosal (2ΔCt)	**IBS**	0.11 (0.07-0.16)	0.13	2.3 (1.3-3.6) x10^-3	0.16	9.0 (6.7-12.9) x10^-4	0.63	3.1 (1.7-5.0) x10^-4	0.13
	**HS**	0.13 (0.08-0.24)		2.7 (1.6-4.2) x10^-3		9.1 (7.3-13.8) x10^-4		4.2 (2.5-6.8) x10^-4	

Abbreviations:

CgA = Chromogranin A   CgB = Chromogranin B   SgII = Secretogranin II   SgIII = Secretogranin III

Supplementary table 2. Correlations (rho) between levels of chromogranins (CgA. CgB) and secretogranins (SgII. SgIII) in fecal and mucosal samples of irritable bowel syndrome patients (IBS, n = 88) and healthy subjects (HS, n = 33)

		Fecal				Mucosal			
**Correlations IBS Granins**		CgA	CgB	SgII	SgIII	CgA	CgB	SgII	SgIII
Fecal	CgA	N.A.	0.86***	0.81***	0.81***	-0.17	-0.09	0.12	-0.07
	CgB	0.86***	N.A.	0.63***	0.78***	-0.22*	-0.12	0.10	-0.05
	SgII	0.81***	0.63***	N.A.	0.64***	-0.04	0.03	0.07	0.02
	SgIII	0.81***	0.78***	0.64***	N.A.	-0.33**	-0.23*	0.24*	-0.23*
Mucosal	CgA	-0.17	-0.22*	-0.04	-0.33**	N.A.	0.74***	-0.02	0.69***
	CgB	-0.09	-0.12	0.03	-0.23*	0.74***	N.A.	0.12	0.75***
	SgII	0.12	0.10	0.07	0.24*	-0.02	0.12	N.A.	-0.07
	SgIII	-0.07	-0.05	0.02	-0.23*	069***	0.75***	-0.07	N.A.
		Fecal				Mucosal			
**Correlations HS Granins**		CgA	CgB	SgII	SgIII	CgA	CgB	SgII	SgIII
Fecal	CgA	N.A.	0.66***	0.75***	0.72***	-0.09	0.09	0.11	-0.14
	CgB	0.66***	N.A.	0.36*	0.47**	-0.02	0.16	0.20	-0.07
	SgII	0.75***	0.36*	N.A.	0.44*	-0.19	0.04	-0.07	-0.12
	SgIII	0.72***	0.47**	0.44*	N.A.	-0.10	0.02	0.30	-0.18
Mucosal	CgA	-0.09	-0.02	-0.19	-0.10	N.A.	0.76***	-0.11	0.89***
	CgB	0.09	0.16	0.04	0.02	0.76***	N.A.	0.15	0.74***
	SgII	0.11	0.20	-0.07	0.30	-0.11	0.15	N.A.	-0.06
	SgIII	-0.14	-0.07	-0.12	-0.18	0.89***	0.74***	-0.06	N.A.

Abbreviations:

CgA = Chromogranin A CgB = Chromogranin B SgII = Secretogranin II SgIII = Secretogranin III

Spearman correlations, * = $p < 0.05$, ** = $p < 0.01$. *** = $p < 0.001$
